# Fundoplication in chronic intractable cough

**DOI:** 10.1186/1745-9974-8-3

**Published:** 2012-07-19

**Authors:** Shoaib Faruqi, Peter Sedman, Warren Jackson, Ian Molyneux, Alyn H Morice

**Affiliations:** 1 Department of Cardiovascular and Respiratory Studies, Castle Hill Hospital, Hull York Medical School, University of Hull, Cottingham, HU16 5JQ, UK; 2 Department of Upper Gastrointestinal and Minimally Invasive Surgery, Castle Hill Hospital, Hull York Medical School, University of Hull, Cottingham, HU16 5JQ, UK; 3 Department of Upper Gastro-Intestinal Physiology, Castle Hill Hospital, Hull York Medical School, University of Hull, Cottingham, HU16 5JQ, UK

**Keywords:** Chronic cough, Reflux, Fundoplication

## Abstract

**Background:**

Airway reflux is a common cause of chronic cough and this is often refractory to medical therapy. Surgery in the form of Nissen fundoplication has been highly successful in the treatment of the classic reflux symptoms of heartburn and dyspepsia. There is a paucity of data regarding response to fundoplication in patients presenting with chronic cough.

**Methods:**

We retrospectively reviewed the case notes of patients from the Hull Cough Clinic who had undergone Nissen fundoplication over the past 6 years. Demographic details, duration of symptoms, presence of other symptoms, results of oesophageal studies, outcome and complications were recorded. Patients were contacted by post and asked to complete a questionnaire detailing current symptoms. In a subgroup with continued troublesome cough 24 hour pharyngeal pH measurements were undertaken.

**Results:**

Forty seven patients underwent fundoplication. The average duration of pre-operative cough was 8 years. Gastro intestinal symptoms were present in the majority. In 30 (64%) patients a positive response to treatment was recorded. Mild dysphagia or bloating was seen in 18 patients following surgery. Four patients needed repeat surgical intervention for modification of fundoplication. One patient developed aspiration pneumonia eight weeks following surgery and died of a myocardial infarction. Two thirds of patients with persisting cough had evidence of airway reflux on pharyngeal pH monitoring.

**Conclusion:**

In these patients with intractable cough a long term response rate of 63% represents a useful therapeutic option. Treatment failure is more frequent than for classic peptic symptoms and may be related to persistent gaseous reflux.

## Introduction

In a number of prospective series gastro-oesophageal reflux disease (GORD) has been demonstrated to be associated with chronic cough. This association has led to the implication that GORD is a causal factor in its pathogenesis. However, classical GORD symptoms of “heart burn” and “dyspepsia” are often absent in patients with other manifestations of airway reflux. Non or weakly acid reflux has also been implicated in the genesis of chronic cough. This suggests that the reflux causing cough is unlike that causing GORD and may be non acidic or even gaseous in nature. Whilst anti-acid medications are effective in treating the classical peptic symptoms of GORD and an empirical therapeutic trial is recommended in guidelines on the management of reflux associated cough the efficacy of this treatment is much more modest [1-3]. This suggests that an alternative treatment strategy aimed at preventing all forms of reflux may have a role.

Surgical treatment of reflux by means of fundoplication, performed using open or laparoscopic techniques, is thought to provide an effective mechanical barrier to gastro-oesophageal reflux and eliminates both acid and non-acid components. A number of studies have shown that fundoplication provides excellent short and long term control of peptic symptoms. The most commonly performed procedure is the Nissen fundoplication [4,5]. This was initially described as an open procedure and the laparoscopic technique has evolved in the past twenty years [6].

Although there are several reports of anti reflux surgery in patients with “atypical” respiratory, or laryngeal symptoms [7-30], very few studies are devoted to patients with chronic cough as the presenting symptom [13,16,23,24]. Limitations of these studies include small numbers and lack of follow up. Here we report the short and long term response in patients with reflux associated chronic cough who underwent laparoscopic Nissen fundoplication. This was a retrospective review and postal survey of patients referred for laparoscopic Nissen fundoplication from the Hull Cough Clinic.

## Methodology

### Patients

All patients undergoing laparoscopic Nissen fundoplication at Hull and East Yorkshire Hospitals Trust from May 2003 to April 2009 who had been referred from the Hull Cough Clinic were identified. Patients had been selected for surgery on the basis of a clinical diagnosis of reflux associated chronic cough using our previously described criteria [31]. Patients had to have failed multiple medical therapeutic trials for reflux associated cough as well as eosinophilic airway disease and rhinitis/post nasal drip. These included acid suppressive therapy, prokinetics, baclofen, inhaled cortico-steroids and first generation antihistamine. Our medical treatment algorithm for reflux associated cough includes 8 week sequential therapeutic trails of acid suppressive therapy (lansoprazole 30 mg bd with ranitidine 300 mg at night), prokinetics (metoclopramide 10 mg tds followed by domperidone 10 mg tds) and baclofen (5 mg tds and increased depending upon response/tolerance to 10 mg tds) for its action on the lower oesophageal sphincter. All patients recruited gave written informed consent for laparoscopic Nissen fundoplication. They had undergone a standard set of pre operative assessments which included 24 hour ambulatory oesophageal pH monitoring and manometry. The pH monitoring studies were done off and acid suppressive therapy. All patients had a chest radiograph which was normal. The case notes of all the patients identified were reviewed in detail by SF. The demographic details, duration of symptoms, prior medical treatment, results of investigations and recorded response to surgery and complications of laparoscopic Nissen fundoplication were elicited. Study approval was obtained from the hospital audit committee and confirmed by the ethics committee of Hull and East Riding, UK.

### Pre-operative assessment

As previously described oesophageal motility was assessed by solid-state manometry [32]. Ambulatory 24 hr oesophageal pH was measured at a level of 5 cm above the pre determined (via oesophageal manometry) upper border of the lower oesophageal sphincter (LOS). Data was presented as DeMeester score or percentage time the pH <4.

### Intervention

Laparoscopic Nissen fundoplication was performed in a standard fashion under general anaesthesia with full muscle relaxation using a five port technique [32]. In every case the oesophageal hiatus was fully dissected and the oesophagus mobilised. At least one non absorbable suture was placed to approximate the crura posterior to the oesophagus and to minimise the risk of post-operative herniation. In cases where there was a large pre-existing hiatal defect, additional posterior crural sutures were placed as required. In most cases there was no obvious hiatus hernia demonstrated at surgery. Calibration of the oesophageal hiatus was clinical but in cases of doubt a 56 Fr endoluminal bougie was available to calibrate the appropriate size.

### Symptom questionnaires

All patients identified were contacted by post in August 2009 and were asked to complete and return a questionnaire. This questionnaire included 100 mm visual analogue scales (VAS) to assess cough and heart burn/indigestion. Patients were asked to indicate on the VAS what they perceived their symptoms were like prior to the surgery and what they were like now (at the time of completing the questionnaire). The scale ranged from “not troubled” to “extremely troubled”. They were similarly also asked to complete the Hull Airway Reflux Questionnaire (HARQ), a self administered airway reflux specific questionnaire [33]. Option to indicate in free text anything else related to either their symptoms, complications or the surgery was also present in the questionnaire.

### Outcomes

The short term outcome was decided upon review of the case notes. Response of cough to laparoscopic Nissen fundoplication was divided into three categories: complete response, partial response and lack of response. Documentation in the notes that the cough had either resolved or ceased to cause any substantial discomfort was categorised as a complete response. If it was documented that there was an improvement in cough but the cough had not completely resolved and was causing significant problems it was categorised as a partial response. Lack of response was documented as such. Similarly the outcomes of “heart burn” and/or “indigestion” if present were recorded.

The long term outcomes were decided based upon the VAS scores for cough from the questionnaires returned. VAS scores for both cough and the peptic symptoms were used.

Complications post procedure were recorded on review of case notes and returned questionnaires.

### Pharyngeal pH monitoring

Pharyngeal airway pH monitoring (Restech) was performed on patients continuing to complain of reflux-associated cough following laparoscopic Nissen fundoplication. A probe is placed in the oropharynx via the nasal route and monitors the pH in the surrounding environment over 24 hours. Sampling frequency (2 Hz) is sufficient to detect short lived, gaseous reflux events which may be responsible for triggering cough. Analysis uses a composite scoring system (Ryan score) based on frequency and duration of episodes where the pH crosses a lower threshold [34]. Studies are scored separately in the upright and supine positions.

### Statistical analysis

Demographics and other applicable data are presented descriptively. The VAS scores for cough and peptic symptoms were analysed by the Wilcoxon signed-rank test. Fischers exact test was performed to look for predictors of successful response of cough to laparoscopic Nissen fundoplication. P value of <0.05 was taken as being significant. Statistical analysis was performed using SPSS statistical software package, Chicago, Illinois, USA.

## Results

Forty seven patients (36 women, median age 55 years) were recruited in this study. The median duration of cough was 5 years (Table 1). Peptic symptoms were documented in the case notes to be present in 85% of the patients. In all the patients cough was the primary indication for laparoscopic Nissen fundoplication. Peptic symptoms by themselves were not an indication for surgery.

**Table 1:** Patient characteristics and outcome

N	47
Women	36
Age (Median, Range)	55 (32–79)
Duration of cough in years (Median, Range)	5.0 (1–30)
Peptic symptoms present in	85%
Significant acid reflux present in	72%
Decreased lower oesophageal sphincter pressure	28%
Response to treatment	64%
Complete	45%
Partial	19%

### Short term outcome

The primary short term outcome of improvement in cough was seen in 30 patients (64%). The response was complete in 45% and partial in 19%. Following surgery peptic symptoms improved in all patients. This is summarised in Table 1.

### Long term outcome

**Figure 1:**
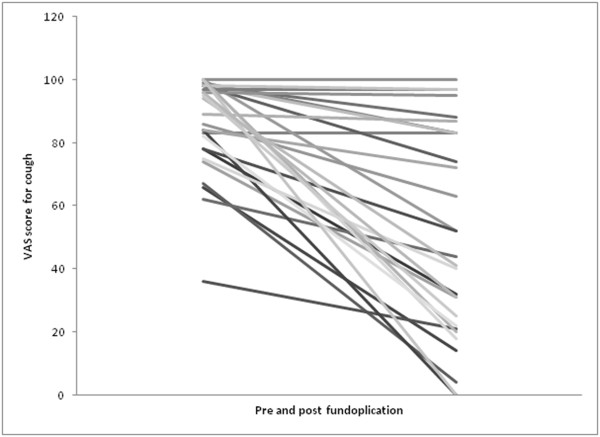
**Individual change in the VAS scores for cough is shown.** The VAS scores are well correlated with the complete response, partial response and lack of response as recorded in the notes.

**Figure 2:**
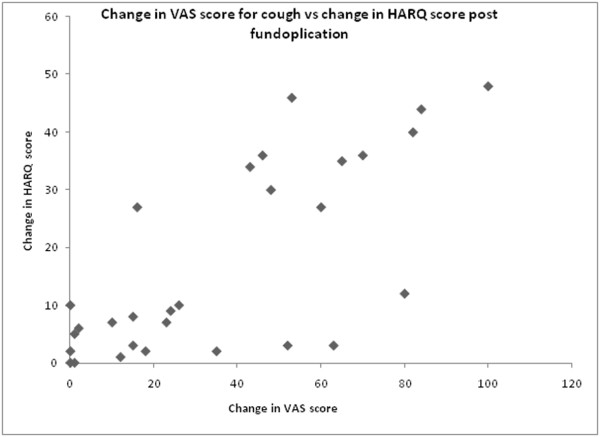
**The change in HARQ score and the VAS score for cough is shown.** The decrease in VAS score for cough correlated well with the decrease in HARQ score following fundoplication (Spearman’s correlation co-efficient = 0.7, p < 0.001).

The questionnaire was returned by 62% of the patients. The mean duration of follow up on return of questionnaire was 3.8 years. The median (range) pre surgery VAS score for cough was 94 (36–100) which decreased to 44 (0–100) at long term follow up (p < 0.001). A similar improvement was seen both in the heart burn/indigestion VAS score as well as the HARQ. These results are summarised in Table 2. Figure 1 demonstrates the individual change in the VAS scores for cough. The VAS scores for cough and the HARQ scores were well correlated with the complete response, partial response and lack of response as recorded in the notes. Only one patient was recorded as having an improvement in his cough when his VAS cough score was unchanged. Three patients were recorded as having an improvement in their dyspeptic symptoms when their VAS score was unchanged. The decrease in VAS score for cough correlated well with the decrease in HARQ score following fundoplication (Spearman’s correlation co-efficient =0.7, p < 0.001). This is shown in Figure 2.

**Table 2:** Long term outcomes

N	29
Median (range) VAS score for cough	
Pre surgery	94 (36–100)
On follow up	44 (0–100), (p < 0.001)
Median (range) VAS score for heart burn/indigestion	
Pre surgery	94 (3–100)
On follow up	16 (0–100), (p < 0.001)
Median (range) VAS HARQ score	
Pre surgery	50 (10–67)
On follow up	28 (0–61), (p < 0.001)

### Predictors of outcome

The factors looked at were gender, presence of heart burn/dyspepsia, abnormal acid reflux on 24 hour pH recording, presence of oesophageal dysmotility and decreased lower oesophageal sphincter tone. None of these parameters were a significant discriminator of outcome.

### Results of pharyngeal pH monitoring

In twelve patients who remained symptomatic post-laparoscopic fundoplication pharyngeal pH studies were performed. Mean HARQ score in this group was 44.2. Eight studies were positive in the upright position (mean Ryan score 83.9, ULN 9.41). All 12 studies were negative in the supine position.

### Complications

Seventeen patients perceived dysphagia following surgery. In 14 the dysphagia was mild and did not need any specific intervention. In 3 patients the Nissen fundoplication needed conversion to a partial (270°) fundoplication and in one patient the fundoplication was reversed. Three patients perceived a sensation of bloating. Two patients complained of discomfort as they were not able to vomit. One patient developed a small left sided pleural effusion post operatively. This resolved without any specific treatment. A 43 year old woman had recurrence of dyspeptic symptoms and cough two years following fundoplication. Dyspeptic symptoms had resolved and cough had improved, the response being partial, following the procedure. On investigation the fundoplication wrap was found to be partially undone. She had a further procedure to ensure effective fundoplication which led to the amelioration of dyspeptic symptoms and improvement in cough, which again was partial. A 69 year old woman had an episode of aspiration pneumonia eight weeks following surgery and suffered fatal myocardial infarction.

## Discussion

We have demonstrated in our study that around two thirds of patients with reflux associated chronic cough have a significant response to Nissen fundoplication. Of the responders two thirds have a very good response with either complete resolution of cough or reduction in cough to levels which were no longer perceived to be a problem. This response was sustained on long term follow up. As suggested by the VAS scores, our patients suffered from debilitating chronic cough which we and others have demonstrated to have a profound impact on quality of life. The post operative reduction in VAS score was not uniform but in those who responded represents a marked improvement. Free text comments written on the questionnaire included “My life was pure hell before the operation”, “The operation changed my life” and “Delighted and would recommend to anyone…”. When successful it is clearly a valuable treatment option.

Reflux has been increasingly implicated in the pathogenesis of cough in patients presenting to specialist cough clinics [3]. Lack of consensus on diagnostic criteria of extra-oesophageal reflux makes it difficult to exactly quantify the association. Conventional criteria based on duration of oesophageal acid exposure observed in twenty four hour pH studies are applicable only to the peptic symptoms of GORD. Impedance measurements demonstrate that non/weakly acid reflux is responsible for many extra-oesophageal symptoms such as cough and dysphonia [3]. Our data suggests that persistent gaseous reflux occurs in patients failing fundoplication. Thus the nature of reflux leading to cough may be different from that causing GORD in that non acid, gaseous refluxate has a leading role.

In the absence of good objective diagnostic tests the clinical history is important in establishing the diagnosis of reflux associated chronic cough. Symptoms which suggest the diagnosis include: cough on phonation, on rising from bed, associated with certain foods or eating [31]. In the validation of our questionnaire, HARQ, designed to detect airway reflux “heart burn” or “indigestion” had the weakest predictive probability [32].

One third of patients had little or no response in terms of cough although improvement in the symptoms of heartburn and regurgitation (data not shown) was almost invariable. The relatively high numbers of patients with reflux related cough having dyspeptic symptoms in our cohort may reflect our bias in selecting those with additional symptoms known to improve with fundoplication. In fact, the presence of dyspepsia did not predict a successful outcome in cough, suggesting different mechanisms underlying the genesis of these symptoms. In patients not responding to Nissen fundoplication pharyngeal pH monitoring demonstrated significant persistent reflux in the majority. Unlike the liquid reflux of GORD which is eliminated by fundoplication the gaseous reflux causing cough may persist.

There is a significant complication rate associated with fundoplication, chief of which are gas bloat and dysphagia. Unfortunately, we were unable to identify factors predicting a successful outcome and so in those patients with isolated cough careful consideration of the risk benefit ratio is required. Laparoscopic Nissen fundoplication can be taken down if there are intolerable complications and no benefit and we have undertaken this in one patient in this cohort.

There have been a number of reports of the experience of fundoplication for a variety of indications including patients with respiratory symptoms. Allen and Anvari reported their cohort of 905 [18]. Of the 209 patients with some respiratory symptoms, 83% had cough. Cough was reported to have improved in 83% at 6 months, 74% at 2 years and 71% at 5 years. Kaufman et al. reported the retrospective long term outcomes in 750 patients who had undergone fundoplication of whom 231 had experienced cough, hoarseness or wheezing once per week [12]. At a median follow up of 53 months there was a durable and statistically significant fall in cough assessed by a symptom questionnaire. Resolution of cough was observed in 41% and improvement in 74%. Very few studies have looked into chronic cough alone as a primary indication for fundoplication. In the largest, a prospective study of 21 consecutive patients undergoing Nissen fundoplication for reflux associated chronic cough complete resolution was observed in 62% and considerable improvement in 76% [23]. Thus the results of our study are very similar to that reported in literature.

Laparoscopic Nissen fundoplication is a relatively safe procedure. In a retrospective review of over 2000 anti-reflux procedures 1 death was observed [25]. Another study of 148 procedures reported peri-operative morbidity and mortality to be 8.8% and 0.7% respectively [19]. Relatively mild complications of Nissen fundoplication are gastro-paresis, dysphagia and bloating. Because of the nature of the procedure a transient degree of dysphagia is experienced. In a majority of patients this improves without the need for any specific intervention. Rarely either a reversal, conversion to a partial fundoplication or oesophageal dilatation is needed.

Our study was retrospective follow up of patients and lacked a control arm. Cough therapy is known to have a strong placebo effect and this cannot be ruled out even in a patient population that had repeatedly failed medical therapy**.** Ideally we would have investigated pharyngeal pH pre and post operatively; however this testing was not clinically indicated and would therefore have required informed consent. This would have also proved prohibitively expensive. We used a VAS score to evaluate response to treatment and this may be subject to a “recall” bias. All other studies evaluating the role of anti-reflux surgery on chronic cough are also uncontrolled and not blinded. They also differ in the basis on which a diagnosis of reflux associated chronic cough and how the outcome was assessed. A randomised controlled trial is urgently needed to fully evaluate this treatment.

## Competing interests

None of the authors have any competing interests relevant to this study.

## Authors’ contributions

SF and AHM designed this study and evaluated patients in the cough clinic for the procedure. SF collected the data and analysed the results. WJ performed the oesophageal and pharyngeal studies. PS is the surgeon who performed the fundoplication for the subjects. IM collected the pharyngeal pH data on the subjects with persistent symptoms following fundoplication and analysed the results of the same. SF wrote the initial draft of the manuscript and conducted the statistical analysis. AHM and SF further edited the manuscript which was approved by all authors. All authors read and approved the final manuscript.

## References

[B1] Morice AH McGarvey L Pavord I British Thoracic Society Cough Guideline Group Recommendations for the management of cough in adults Thorax 2006 61 Suppl 1 i1 i24 16936230 10.1136/thx.2006.065144 PMC2080754

[B2] Morice AH Fontana GA Sovijarvi AR Pistolesi M Chung KF Widdicombe J O'Connell F Geppetti P Gronke L De Jongste J Belvisi M Dicpinigaitis P Fischer A McGarvey L Fokkens WJ Kastelik J ERS Task Force The diagnosis and management of chronic cough Eur Respir J 2004 24 3 481 492 10.1183/09031936.04.00027804 15358710

[B3] Chandra KM Harding SM Therapy Insight: treatment of gastroesophageal reflux in adults with chronic cough Nat Clin Pract Gastroenterol Hepatol 2007 4 11 604 613 10.1038/ncpgasthep0955 17978817

[B4] Broeders JA Draaisma WA Bredenoord AJ Smout AJ Broeders IA Gooszen HG Long-term outcome of Nissen fundoplication in non-erosive and erosive gastro-oesophageal reflux disease Br J Surg 2010 97 6 845 852 10.1002/bjs.7023 20473997

[B5] Pessaux P Arnaud JP Delattre JF Meyer C Baulieux J Mosnier H Laparoscopic antireflux surgery: five-year results and beyond in 1340 patients Arch Surg 2005 140 10 946 951 10.1001/archsurg.140.10.946 16230543

[B6] Dallemagne B Weerts JM Jehaes C Markiewicz S Lombard R Laparoscopic Nissen fundoplication: preliminary report. Surg Laparosc Endosc 1991 1 3 138 143 1669393

[B7] Hamdy E El-Shahawy M Abd El-Shoubary M Abd El-Raouf A El-Hemaly M Salah T El-Hanafy E GadEl Hak N Response of atypical symptoms of GERD to antireflux surgery Hepatogastroenterology 2009 56 90 403 406 19579608

[B8] Iqbal M Batch AJ Moorthy K Cooper BT Spychal RT Outcome of surgical fundoplication for extra-oesophageal symptoms of reflux Surg Endosc 2009 23 3 557 561 10.1007/s00464-008-9861-8 18365279

[B9] Catania RA Kavic SM Roth JS Lee TH Meyer T Fantry GT Castellanos PF Park A Laparoscopic Nissen fundoplication effectively relieves symptoms in patients with laryngopharyngeal reflux J Gastrointest Surg 2007 11 12 1579 1587 10.1007/s11605-007-0318-5 17932726

[B10] Ranson ME Danielson A Maxwell JG Harris JA Prospective study of laparoscopic Nissen fundoplication in a community hospital and its effect on typical, atypical, and nonspecific gastrointestinal symptoms JSLS 2007 11 1 66 71 17651559 PMC3015813

[B11] Salminen P Sala E Koskenvuo J Karvonen J Ovaska J Reflux laryngitis: a feasible indication for laparoscopic antireflux surgery? Surg Laparosc Endosc Percutan Tech 2007 17 2 73 78 10.1097/SLE.0b013e31803bb500 17450083

[B12] Kaufman JA Houghland JE Quiroga E Cahill M Pellegrini CA Oelschlager BK Long-term outcomes of laparoscopic antireflux surgery for gastroesophageal reflux disease (GERD)-related airway disorder Surg Endosc 2006 20 12 1824 1830 10.1007/s00464-005-0329-9 17063301

[B13] Tutuian R Mainie I Agrawal A Bloomston M Albrink M Goldin S Rosemurgy A Nonacid reflux in patients with chronic cough on acid-suppressive therapy Chest 2006 130 2 386 391 10.1378/chest.130.2.386 16899836

[B14] Rakita S Villadolid D Thomas A Bloomston M Albrink M Goldin S Rosemurgy A Laparoscopic Nissen fundoplication offers high patient satisfaction with relief of extraesophageal symptoms of gastroesophageal reflux disease Am Surg 2006 72 3 207 215 16553119

[B15] Liu JJ Carr-Locke DL Osterman MT Li X Maurer R Brooks DC Ashley SW Saltzman JR Endoscopic treatment for atypical manifestations of gastroesophageal reflux disease Am J Gastroenterol 2006 101 3 440 445 10.1111/j.1572-0241.2006.00496.x 16542278

[B16] Ziora D Jarosz W Dzielicki J Ciekalski J Krzywiecki A Dworniczak S Kozielski J Citric acid cough threshold in patients with gastroesophageal reflux disease rises after laparoscopic fundoplication Chest 2005 128 4 2458 2464 10.1378/chest.128.4.2458 16236909

[B17] Mainie I Tutuian R Agrawal A Hila A Highland KB Adams DB Castell DO Fundoplication eliminates chronic cough due to non-acid reflux identified by impedance pH monitoring Thorax 2005 60 6 521 523 10.1136/thx.2005.040139 15923255 PMC1747420

[B18] Allen CJ Anvari M Does laparoscopic fundoplication provide long-term control of gastroesophageal reflux related cough? Surg Endosc 2004 18 4 633 637 10.1007/s00464-003-8821-6 15026893

[B19] Duffy JP Maggard M Hiyama DT Atkinson JB McFadden DW Ko CY Hines OJ Laparoscopic Nissen fundoplication improves quality of life in patients with atypical symptoms of gastroesophageal reflux Am Surg 2003 69 10 833 838 14570358

[B20] Brouwer R Kiroff GK Improvement of respiratory symptoms following laparoscopic Nissen fundoplication ANZ J Surg 2003 73 4 189 193 10.1046/j.1445-1433.2002.02568.x 12662224

[B21] Allen CJ Anvari M Preoperative symptom evaluation and esophageal acid infusion predict response to laparoscopic Nissen fundoplication in gastroesophageal reflux patients who present with cough Surg Endosc 2002 16 7 1037 1041 10.1007/s00464-001-8330-4 12165818

[B22] Thoman DS Hui TT Spyrou M Phillips EH Laparoscopic antireflux surgery and its effect on cough in patients with gastroesophageal reflux disease J Gastrointest Surg 2002 6 1 17 21 10.1016/S1091-255X(01)00013-0 11986013

[B23] Novitsky YW Zawacki JK Irwin RS French CT Hussey VM Callery MP Chronic cough due to gastroesophageal reflux disease: efficacy of antireflux surgery Surg Endosc 2002 16 4 567 571 10.1007/s00464-001-8328-y 11972189

[B24] Irwin RS Zawacki JK Wilson MM French CT Callery MP Chronic cough due to gastroesophageal reflux disease: failure to resolve despite total/near-total elimination of esophageal acid Chest 2002 121 4 1132 1140 10.1378/chest.121.4.1132 11948043

[B25] Greason KL Miller DL Deschamps C Allen MS Nichols FC 3rd Trastek VF Pairolero PC Effects of antireflux procedures on respiratory symptoms Ann Thorac Surg 2002 73 2 381 385 10.1016/S0003-4975(01)03407-5 11845846

[B26] Farrell TM Richardson WS Trus TL Smith CD Hunter JG Response of atypical symptoms of gastro-oesophageal reflux to antireflux surgery Br J Surg 2001 88 12 1649 1652 10.1046/j.0007-1323.2001.01949.x 11736981

[B27] Ekström T Johansson KE Effects of anti-reflux surgery on chronic cough and asthma in patients with gastro-oesophageal reflux disease Respir Med 2000 94 12 1166 1170 10.1053/rmed.2000.0944 11192951

[B28] Chen RY Thomas RJ Results of laparoscopic fundoplication where atypical symptoms coexist with oesophageal reflux Aust N Z J Surg 2000 70 12 840 842 10.1046/j.1440-1622.2000.01981.x 11167570

[B29] Patti MG Arcerito M Tamburini A Diener U Feo CV Safadi B Fisichella P Way LW Effect of laparoscopic fundoplication on gastroesophageal reflux disease-induced respiratory symptoms J Gastrointest Surg 2000 4 2 143 149 10.1016/S1091-255X(00)80050-5 10675237

[B30] Allen CJ Anvari M Gastro-oesophageal reflux related cough and its response to laparoscopic fundoplication Thorax 1998 53 11 963 968 10.1136/thx.53.11.963 10193396 PMC1745120

[B31] Everett CF Morice AH Clinical history in gastroesophageal cough Respir Med 2007 101 2 345 348 10.1016/j.rmed.2006.05.006 16787744

[B32] Fathi H Moon T Donaldson J Jackson W Sedman P Morice AH Cough in adult cystic fibrosis: diagnosis and response to fundoplication Cough 2009 5 1 10.1186/1745-9974-5-1 19149907 PMC2634760

[B33] Morice AH Faruqi S Wright CE Thompson R Bland JM Cough hypersensitivity syndrome: a distinct clinical entity Lung 2011 189 1 73 79 10.1007/s00408-010-9272-1 21240613

[B34] Ayazi S Lipham JC Hagen JA Tang AL Zehetner J Leers JM Oezcelik A Abate E Banki F DeMeester SR DeMeester TR A new technique for measurement of pharyngeal pH: normal values and discriminating pH threshold J Gastrointest Surg 2009 13 8 1422 1429 10.1007/s11605-009-0915-6 19421822

